# Albumin Nanostructures for Nucleic Acid Delivery in Cancer: Current Trend, Emerging Issues, and Possible Solutions

**DOI:** 10.3390/cancers13143454

**Published:** 2021-07-09

**Authors:** Rama Prajapati, Álvaro Somoza

**Affiliations:** Instituto Madrileño de Estudios Avanzados en Nanociencia (IMDEA Nanociencia), Faraday 9, 28049 Madrid, Spain; rama.prajapati@imdea.org

**Keywords:** albumin, gene therapy, cancer, nanocarriers, surface modification

## Abstract

**Simple Summary:**

Nucleic acids are showing tremendous potential in cancer therapy. However, their successful delivery to tumor sites is still a challenge. Herein, we report on the use of albumin-based nanocarriers for the delivery of nucleic acids because of their biosafety, ease of surface modification, and tumor targeting. In addition, we discuss various surface modification strategies to improve the internalization, efficacy, and specific tumor targeting of the albumin nanocarriers.

**Abstract:**

Cancer is one of the major health problems worldwide, and hence, suitable therapies with enhanced efficacy and reduced side effects are desired. Gene therapy, involving plasmids, small interfering RNAs, and antisense oligonucleotides have been showing promising potential in cancer therapy. In recent years, the preparation of various carriers for nucleic acid delivery to the tumor sites is gaining attention since intracellular and extracellular barriers impart major challenges in the delivery of naked nucleic acids. Albumin is a versatile protein being used widely for developing carriers for nucleic acids. It provides biocompatibility, tumor specificity, the possibility for surface modification, and reduces toxicity. In this review, the advantages of using nucleic acids in cancer therapy and the challenges associated with their delivery are presented. The focus of this article is on the different types of albumin nanocarriers, such as nanoparticles, polyplexes, and nanoconjugates, employed to overcome the limitations of the direct use of nucleic acids in vivo. This review also highlights various approaches for the modification of the surface of albumin to enhance its transfection efficiency and targeted delivery in the tumor sites.

## 1. Introduction

Cancer is one of the major public health problems and a leading cause of morbidity and mortality worldwide [1,2]. According to the data presented in Cancer Statistics, 2020, the 5-year relative survival rate for all cancers diagnosed from 2009 to 2015 was 67% [2]. Despite being one of the major causes of death, early tumor diagnosis and efficient therapy are still a challenge. The current cancer therapy includes surgical intervention, radiation therapy, and chemotherapy with the aim of tumor shrinking and cancer relapse reduction. However, chemotherapy is often associated with side effects caused by the off-site toxicity due to the lack of drug specificity [3]. Therefore, the design of more efficient therapies with improved selectivity to the tumor sites is desired. 

Currently, gene therapy in cancer is gaining increasing scientific and clinical interest because of various revelations regarding the origin of cancers from genetic errors, either environmentally triggered or hereditary. Gene therapy is aimed at treating or repairing the errors occurring in tumor suppressor genes, oncogenes, or DNA pathways by substitution or addition of a functional gene into the living cell [4]. However, its success is challenged by the high molecular weight, enzymatic degradation, and anionic nature of nucleic acids [5,6]. In this regard, nanostructures are gaining increasing popularity as nucleic acids delivery vehicles due to low off-target effects, improvement of current therapies, and protection of nucleic acids from enzymatic degradation [7,8]. By modulating the chemical and physical properties of nanostructures, their biological characteristics, including cellular uptake, toxicity, immunogenicity, and efficacy, can be regulated [7,9]. Moreover, nanostructures can be accumulated in the tumor sites due to leaky vessels caused by rapid and excessive angiogenesis, commonly known as the enhanced permeability and retention (EPR) effect [10]. In addition to passive targeting by the EPR effect, the active targeting can be achieved through the use of different targeting moieties, such as antibodies, aptamers, or small molecules that interact with great selectivity with selected receptors in the cell surface [11,12,13]. 

In the case of the application of gene therapy in cancer, nanocarriers based on polymers, lipids, and metals are widely being investigated. However, their clinical application is limited because of their toxicity, scale-up complications, and immunogenicity [9]. In this regard, protein-based nanocarriers have shown promising use in cancer because of their unique features such as biocompatibility, safety, tumor targeting by surface modification, ease of preparation, and broad stability profiles. Taking into consideration the above-mentioned facts, the current review is focused on the albumin-based nanostructures for the delivery of nucleic acids in cancer therapy. We summarize the challenges associated with the systemic delivery of nucleic acids and discuss how albumin-based nanocarriers can overcome these obstacles. In addition, we highlight the current issues with albumin-based systems and several approaches to overcome those challenges by modifying the surface to improve the therapeutic efficacy and targeted gene delivery.

## 2. Nucleic Acids in Cancer Therapy

Gene therapy considers the molecular basis of the diseases and refers to the transfer of genetic material into cells with the aim of a therapeutic response. The first human in-vivo gene transfer study was conducted by Rosenberg and co-workers in 1990 in patients with advanced melanoma [14]. The study showed the feasibility, safety, and potency of using gene therapy in humans. This finding has revolutionized the field of gene therapy and from the last two decades, multiple approaches have confirmed the potential of nucleic acids for the treatment of various types of cancer [15,16]. The most widely used nucleic acids for cancer therapy include small interfering RNA (siRNA), antisense oligonucleotides (ASOs), aptamers, micro RNAs (miRNA), and plasmid DNA (pDNA) [17,18]. Their mechanism of action varies widely, ranging from mRNA regulation to protein binding, which can be designed to promote the reduction in cancer cell proliferation, induction of apoptosis, enhancement of immune-stimulatory responses, and inhibition of neoangiogenesis [19,20,21]. The small RNAs form RNA-induced silencing complex (RISC), which in turn silences the mRNA translation, whereas ASOs can act either by suppression of the ribonucleoprotein activity or by activation of the enzymatic cascade that enhances mRNA degradation [22]. The great therapeutic potential of nucleic acids has been assessed in multiple experiments in cell culture or animal models [23,24]. However, some challenges need to be addressed to ease their path to the clinic.

### 2.1. Limitations Associated with Nucleic Acid Delivery

**Figure 1:**
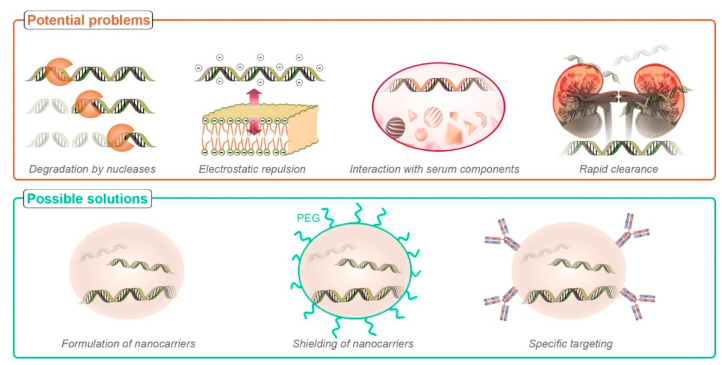
Various barriers to nucleic acid delivery and their possible solutions.

Despite the promising therapeutic applications of nucleic acids in cancer therapy, their effective delivery to the target sites is still challenging [25,26]. Particularly, the major drawbacks associated with nucleic acid delivery include difficulty in accessing deeply seated tumor sites, biological barriers, enzymatic degradation by nucleases, rapid clearance by kidney filtration, triggering of the immune system, and effects in non-targeted genes. The naked nucleic acids cannot enter the cells because of their inherent properties such as hydrophilicity, large size, and negative charges [6]. Nucleic acids are rapidly degraded by intra- and extracellular enzymes even before reaching the target cells. This was demonstrated in a study conducted in mice where a pDNA was fully degraded within 5 min after the injection [27]. Hence, the sufficient genes required to elicit the therapeutic effect cannot be reached at the target sites. The nucleic acids can also trigger the immune system resulting in the release of cytokines that may further lead to serious inflammatory responses [28]. In addition, inhibition or overexpression of a non-targeted gene, commonly called the “off-target” effect, is one major setback in nucleic acid therapy [29]. The major limitations associated with the delivery of nucleic acids are demonstrated in Figure 1 along with the possible solutions.

### 2.2. Nanocarriers for Nucleic Acid Delivery

To overcome the challenges associated with the delivery of nucleic acids to cancer sites, viral and non-viral vectors are being used extensively. The viral vectors show high transfection efficiency and possess strong promoters for the long-term expression of genes. However, carcinogenicity, inflammation, immunogenicity, and high production costs are still the primary concerns [30]. On the other hand, non-viral vectors based on lipids, proteins, and polymers are considered relatively safe but show low and transient gene transfection. Hence, the development of biocompatible and biodegradable delivery vehicles possessing specificity to the target sites, and avoiding immune system activation is of utter importance [5]. In this regard, protein-based nanocarriers are gaining much popularity [31].

## 3. Albumin-Based Nanocarriers

### 3.1. Albumin

Albumin, with a molecular weight of around 67 kDa is the most abundant protein in human blood, which is synthesized in the liver and has a circulation half-life of approximately 19 days [32]. Albumin has an overall negatively charged surface, which makes it highly water-soluble [33]. It has various ligand binding sites, namely Sudlow’s site I, which mainly binds the dicarboxylic acids and bulky heterocyclic molecules and, Sudlow´s site II (indole-benzodiazepine site), which has an affinity towards the aromatic carboxylic acids [34]. The high stability of albumin is attributed to the disulfide bonds formed internally by 34 cysteine residues [35]. In addition, it has one free cysteine residue on the outer surface, which is responsible for the conjugation of ligands [32,35]. Albumin transcytosis is mediated by various receptors such as GP60, also known as albondin, SPARC, also known as osteonectin, GP18, and GP30. GP18 and GP30 receptors are mostly responsible for the lysosomal degradation of deleterious albumin since these receptors have an affinity to the modified albumin such as oxidized or glycated ones [32,36]. The unique properties of albumin, including long half-life, the ability of cellular receptor-mediated transcytosis, and surface properties aiding in the conjugation of other moieties, make it a suitable candidate for the preparation of nanocarriers. In this sense, the most commonly used albumins include ovalbumin, bovine serum albumin (BSA), and human serum albumin (HSA) [37]. Among them, BSA is most widely accepted because of its low cost, abundance, and ease of purification, whereas HSA is used to avoid any immunological response in studies involving humans [37].

### 3.2. Albumin in Cancer Therapy

**Figure 2:**
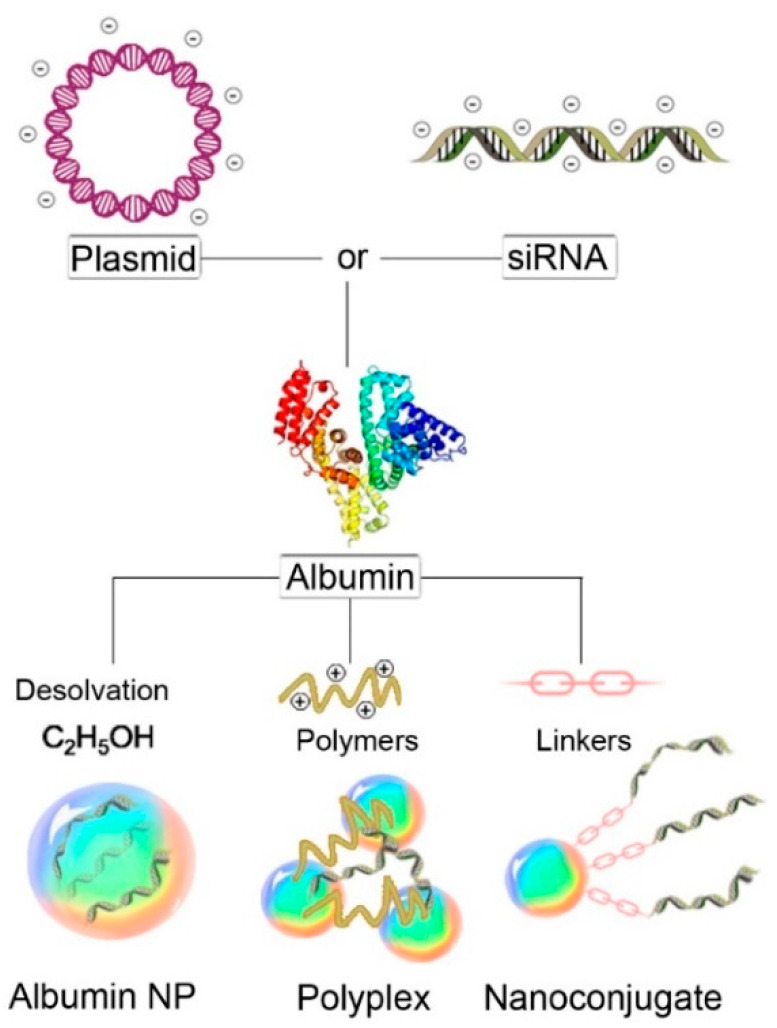
Schematic representation of different albumin nanocarriers for gene therapy.

Albumin is being investigated extensively in cancer therapy due to its excellent properties as a selective carrier in this type of disease. This is due to many factors that lead to a preferable accumulation of the albumin structures in the tumor. For instance, the high concentration of albumin in the blood (40 mg/mL) compared to the interstitial concentration of 14 mg/mL aids in the diffusional transport of albumin to tumor sites [38,39]. In addition, albumin is preferentially internalized as the source of amino acids to cope with the enhanced cellular growth by the cancer cells expressing oncogenic Ras, whose activation is associated with cancer [33]. This property can be utilized to deliver the cargo encapsulated in albumin to cancer cells. Moreover, the albumin-binding proteins, namely gp60 and SPARC, are overexpressed in the cancer cells, which provides specificity to targeting the tumor sites [40]. The protein Cav-1 responsible for the formation of caveolae is upregulated in cancer cells, and since endocytosis of albumin is mainly mediated through caveolae, the accumulation of albumin in cancer sites is further enhanced [33,41]. Albumin is hence being used in pharmaceutical applications as a biocompatible and biodegradable carrier for the delivery of anti-cancer agents, such as chemotherapeutics, biologics, and immunomodulatory drugs. So far, the most studied albumin-based delivery systems for nucleic acids are nanoparticles, nanoconjugates and polyplexes (Figure 2).

### 3.3. Albumin Nanocarrier for Gene Therapy in Cancer 

In comparison to other nanocarriers, albumin-based nanocarriers provide various advantages including easy and reproducible production, possible scale-up options, and in addition, do not show undesired interaction with the serum [42,43]. Considering those advantages and its success in the delivery of chemotherapeutic agents, serum albumin can also be utilized for the delivery of nucleic acids. A wide range of studies on albumin nanocarriers have been conducted to efficiently deliver various genetic materials to the tumor sites (Table 1). In addition, albumin-based nanocarriers are finding their promising application in cancer immunotherapy in recent years. 

### 3.4. Albumin Nanoparticles 

One of the most widely used methods of utilizing albumins (e.g., BSA, HSA) as a carrier for nucleic acids in cancer therapy is by encapsulation of the desired nucleic acids into albumin-based nanoparticles [54]. These structures can be prepared by various techniques, including desolvation, thermal gelation, emulsification, nanospray drying, and self-assembly. Among all those methods, desolvation is the most practiced method using ethanol as a desolvating agent and glutaraldehyde as a cross-linker [16,55,56]. The albumin nanoparticles protect the integrity of encapsulated nucleic acids and prevent their enzymatic degradation. They enter the cells via an energy-dependent mechanism, primarily through caveolae- and clathrin-mediated endocytotic pathways [55]. Albumin nanoparticles have been employed to deliver different nucleic acids, such as plasmids, oligonucleotides, and siRNAs, as detailed below.

#### 3.4.1. Plasmid

A plasmid is a double-stranded DNA molecule (few hundreds to thousands of base pairs (bp)), which is usually circular and contains the transgene encoding for specific proteins [57]. Albumin-based nanoparticles are emerging as suitable candidates for plasmid delivery in cancer therapy because of the high efficiency of DNA transfection into tumor cells, non-toxicity, and biodegradability imparted by albumin molecules. Albumin nanoparticles loaded with plasmids have shown anti-tumor efficacy in various cancers, including breast, pancreatic, brain, and lung [23,24,58,59]. For instance, BSA nanoparticles encapsulating the hMDA-7 plasmid, which encodes for the melanoma differentiation-associated gene, were used against pancreatic cancer [24]. This formulation was used in BXPC-3 cells, inducing an apoptosis rate of 25.6 compared to 15.3 obtained through the direct addition of the plasmid. The system was also assessed in mouse xenografts where the tumor growth was suppressed and the downregulation of VEGF, MMP-2, and MMP-9 proteins was also observed.

Albumin nanostructures have also been used in combination with other nanoparticles, such as magnetic nanoparticles (MNPs), where the albumin nanospheres wrap the MNPs. This formulation enhances the biocompatibility of MNPs, due to the presence of albumin. Furthermore, the presence of MNPs allows for better control of the release of encapsulated genes since it can be triggered by magnetic hyperthermia, and also, the accumulation of the nanoparticles in the tumor can be enhanced through magnetic targeting [59,60]. In this regard, Hou and co-workers employed iron oxide SPIONs encapsulated in albumin nanospheres to deliver survivin-shRNA plasmid encoding short hairpin RNA (shRNA). The system was precisely delivered to the tumor cells in the lung by placing a magnet close to the tumor (magnetic targeting) [59]. In another study, Zhang and co-workers evaluated the combination of the delivery of a plasmid encoding for IFNγ with radiation therapy [60]. The remarkable antitumoral effect obtained highlights the potential of albumin nanocarriers for gene therapy in combination with other therapies, namely radiotherapy, magnetic field stimulated targeting, and molecular targeted therapy, for the treatment of cancer.

#### 3.4.2. Oligonucleotides

Other nucleic acids delivered by albumin nanoparticles are oligonucleotides, which are short (ca. 8–40 nucleotides), single-stranded DNA or RNA molecules. These structures can be used to modulate the gene expression selectively by the inhibition of mRNA processing and its translation. When used for this purpose, they are known as antisense oligonucleotides (ASOs). They can be used to target disease-related genes, including those involved in cancer [47,61]. However, their use in vivo is challenged by their rapid renal clearance and low membrane permeability owing to their size and polyanionic backbone [47]. To overcome these limitations, albumin-based nanoparticles are being explored since they can form stable complexes due to the sequence-independent interaction between oligonucleotides and site I of albumin [62]. In this regard, Wartlick and co-workers described the use of HSA to generate nanoparticles loaded with ASOs, which resulted in enhanced cellular internalization in MCF-7 and MDA-MB-435 cells without significant cytotoxicity [47]. Another study reported the efficient delivery of Akt1 ASOs in KB cells and A549 cells using albumin nanoparticles containing a folate derivative [48].

#### 3.4.3. siRNA

Another type of nucleic acid used in the regulation of gene expression is the small interfering RNAs (siRNAs). These RNAs can recognize a homologous mRNA sequence in a selective and sequence-dependent manner and induce gene silencing in a very efficient manner due to the formation of a catalytic complex with a protein known as RISC [63,64]. Despite their excellent inhibition activity, they are very labile, and for this reason, the use of carriers is an excellent means to increase their overall stability in biological media and improve their efficacy. In this regard, albumin nanoparticles have been used for the delivery of siRNAs to a variety of tumor cells [49,50], where the internalization can be significantly improved through transcytosis [65]. Son and co-workers [49] exploited this approach through the introduction of thiol moieties in both HSA and VEGF siRNAs followed by the formation of stable nanoparticles by self-crosslinking. In this case, the presence of thiol groups in the siRNAs and albumin improved binding affinity to each other and led to a more robust structure. These albumin-based nanoparticles presented excellent properties in PC-3 xenograft models, where the nanoparticles accumulated in the tumoral area, and lead to a significant reduction in tumor volume (80%) [49]. On the other hand, free thiolated siRNAs were rapidly excreted through the kidneys, preventing their accumulation in the tumor.

### 3.5. Polyplexes

Another type of nanostructure based on albumin employed in the delivery of nucleic acids are polyplexes. These structures contain positively charged polymers that interact with the negatively charged nucleic acids, inducing their condensation into smaller structures. The formation of this complex protects the nucleic acids against degradation by nucleases and also increases their internalization, since the positive charges present in the surface of the nanoparticle interact with the negatively charged cell membranes [66,67]. Despite the excellent properties reported for the transfection of nucleic acids, they present some toxicity, which has motivated the search for complementary transfection systems or additives to mitigate this drawback. In this regard, several studies have reported that albumin can enhance the transfection efficiency of polyplexes and improve cell viability [44,68]. 

For instance, in a study conducted by Syga and co-workers, the use of albumin in a PEI-pDNA polyplex accelerated and enhanced the transfection in HeLa cells [44]. They prepared two types of polyplexes, Type 1, where BSA was placed between the plasmid pGFP and PEI, and Type 2 where albumin was added at the end, on the surface of previously formed polyplexes (PEI + pGFP). The experiments revealed that transfection efficiency was better with Type 1 polyplexes as the release of plasmid was easier from the loosely formed polyplexes compared to the Type 2 polyplexes with strong interaction between PEI and plasmid. Similarly, in a study conducted by Nicoli and co-workers, enhancement in cellular uptake was observed in metastatic breast cancer epithelial cells when HSA was incorporated in branched polyethylenimine (bPEI)-siRNA polyplexes [69].

### 3.6. Nanoconjugates

Albumin nanoconjugates are obtained by the interaction of albumin with other moieties such as polymers, nucleic acids, or metals. The interaction may be either non-covalent (hydrophobic and electrostatic) or covalent (thiol-maleimide coupling, Michael addition reaction, and carbodiimide coupling reactions) [70]. Nanoconjugates are smaller (~10 nm) than the typical nanoparticles (~100 nm) and can overcome the limitations associated with the nanoparticles, such as limited biodistribution and toxicity [71]. However, these small conjugates are rapidly metabolized, excreted in vivo, and less effective in exploiting the EPR effect to reach the tumor sites than conventional nanoparticles [72].

In a study conducted by Carver and co-workers, HSA nanoconjugates with RGD-623 oligonucleotides having a size of about 13 nm were prepared [73]. Interestingly, the resulting HSA-RGD-623 conjugate could penetrate a 3D tumor spheroid, whereas the conventional nanoparticles could deliver their payload only on the exterior cells of the spheroid, limiting the induction of splice correction of both GFP654 and Luc705 reporter genes. Similarly, in a study by Sarett and co-workers, serum albumin was used as a carrier in vivo for siRNAs modified with a diacyl lipid moiety (siRNA-L_2_), which enhanced the pharmacokinetic properties of siRNA. This nanoconjugate showed 19-fold more tumor accumulation and 46-fold cellular uptake compared to the commercial siRNA nanocarrier jetPEI, in a mouse orthotopic model of human triple-negative breast cancer [74]. Despite the various advantages of modifying the nucleic acids to increase the stability, pharmacokinetics and pharmacodynamic properties, and enhancement of internalization and endosomal escape, limited work has been done using albumin nanocarriers [75]. Further studies integrating the advantages of albumin nanocarriers with the modified nucleic acids can be of great potential in cancer therapy. 

## 4. Albumin as a Coating Agent

Besides its use as a nanocarrier, albumin can be used as a coating agent for a variety of nanostructures, thus the advantages mentioned before on the use of albumin can be implemented to other nanostructures [51,76,77]. In a study conducted by Xu and co-workers, a chitosan complex with siRNAs was coated with pH-responsive detachable BSA to enhance recognition by human hepatocellular carcinoma cells and suppression of tumor cell proliferation [77]. In this case, the mRNA silencing obtained by the chitosan NPs was improved from 46.9% to 61.8% by the introduction of a BSA coating.

Albumin has been used as a coating agent in various lipid-based nanocarriers, to minimize their interaction with serum proteins and improve their delivery to the target sites [51,78]. For instance, HSA was used to coat lipid nanoparticles loaded with siRNA targeted against GFP (HSA-LNPs-siRNA) and their activity was evaluated in breast cancer cells and the corresponding xenograft mouse model [51]. In the cell experiments, the nanoparticles containing HSA significantly reduced the GFP fluorescence, compared to uncoated lipid nanoparticles. This result was also obtained in the animal model, where a 37% reduction in the GFP expression was achieved after systemic administration of the HSA-coated nanoparticles. In another study, HSA was employed to coat lipid nanoparticles loaded with an antisense oligonucleotide against Bcl-2, which were evaluated in KB human oral carcinoma cells [78]. Interestingly, the authors reported that the efficiency of the Bcl-2 down-regulation depended on the molar ratio of HSA employed. The optimum down-regulation was observed with an HSA to liposome ratio of 3:100 after which the increment in HSA decreased the efficiency.

## 5. Nucleic Acid-Loaded Albumin Nanocarriers for Immunotherapy

Cancer immunotherapy aims to exploit the patients’ own immune systems to treat cancer. Some of the approaches to cancer immunotherapy include immune checkpoint blockade, cancer vaccines, adoptive cell transfer therapy, and oncolytic virotherapy [79]. Among all, immune checkpoint inhibitors have gained wide success in cancer treatment, however, only a limited number of patients benefit from these therapies, where the induction of resistance and toxicity are still huge problems [80]. Interestingly, nucleic acid therapeutics are emerging as the potential candidate for cancer immunotherapy, which may improve the therapeutic outcome in a wide range of tumors, and even in the late stages [81]. These nucleic acids include immunostimulatory DNA/RNA, genome editing nucleic acids, and mRNA/plasmid, which can be further translated to immunotherapeutic proteins [82]. In addition, different genetic tools such as gene editing, gene silencing, or gene activating systems are also being studied extensively in cancer immunotherapy [81]. Nonetheless, despite the tremendous potential of nucleic acids in cancer immunotherapy, the major limitation in the implementation of these techniques in clinical practice is the lack of an efficient delivery vehicle targeted to the cancer cells. In this context, albumin-based nanocarriers are being investigated in a variety of cancers. For instance, Cheng and co-workers developed HSA NPs complexed with stearyl PEI (*st*PEI), which was non-covalently bound to plasmid (CRISPR/Cas9) and a siRNA that silenced the expression of programmed cell death ligand-1 (PD-L1) for cancer immunotherapy [53]. This combined approach produced a synergistic effect where the PD-L1 expression was inhibited by 21.2%.

In summary, immunotherapy against cancer mediated by nucleic acids has enormous potential, as highlighted by the recent developments, such as chimeric antigen receptors (CARs), to treat leukemia (e.g., Kymriah [83]), or CRISPR/Cas9 approaches employed to enhance T-cell mediated gene therapy [84]. However, such systems can be further improved by nanocarriers, such as those based on albumin. 

## 6. Emerging Issues and Possible Solutions

**Figure 3:**
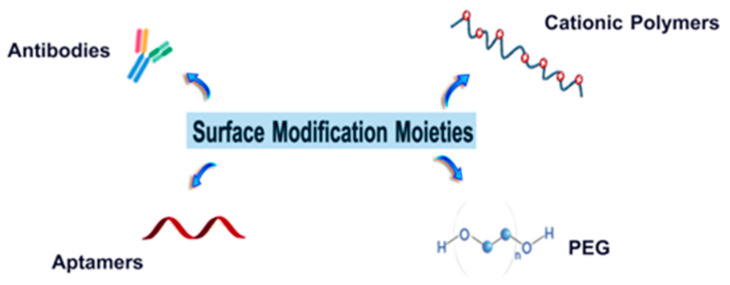
Various moieties for modifying the surface of albumin.

Despite the multiple advantages reported on the use of albumin as a carrier of nucleic acids, there are still several limitations that reduce the efficiency of the process, most of them due to its negative charge at physiological conditions that prevent the binding of anionic nucleic acids [85,86]. Also, other parameters such as the circulation time, specific site targeting, internalization, and release of encapsulated cargo, might require some improvement to ease their clinical translation [87]. In this regard, the modification of albumin-based nanostructures with selected moieties seems to be the most suitable approach [88]. The availability of functional groups such as carboxyl (e.g., asparaginic, glutaminic acid), amino (e.g., lysine), and hydroxyl groups (e.g., tyrosine) on albumin ease the surface modification of the nanostructures. In this sense, some of the common moieties (polymers and targeting agents) employed for surface modification (Figure 3) are discussed below.

### 6.1. Polymers

#### 6.1.1. Cationic Polymers

These polymers are usually employed to improve the binding efficiency of albumin-based nanocarriers to nucleic acids, which are negatively charged. The most common polymers are polyethylenimine (PEI), poly-L-lysine (PLL), and polylactide-co-glycolide (PLGA). In this regard, the introduction of a lysine-based polymer increased the complexation of siRNAs from 16% to 53% [89]. This is presumably because the cationic polymer prevents the siRNA from diffusion during the preparation step and helps in retaining within the NPs. In a study conducted by Rhaeese and co-workers, HSA nanoparticles coated with polyethylenimine (PEI) were employed to efficiently deliver a plasmid DNA in human embryonic epithelial kidney 293 (HEK293) cells [45]. In a similar way, siRNAs were also delivered in MCF-7 breast cancer cells [90].

However, cytotoxicity associated with cationic polymers is still a major concern despite improved transfection efficiency, and hence improving the cell viability while using cationic polymer should be considered. Several strategies are being assessed to overcome this challenge, such as the use of low molecular weight polymers linked with disulfide linkage, fluorinated polymers, PEGylation, or degradable polymers [91,92,93]. The use of these hydrophilic groups enhances the serum stability and circulation times in the blood. In a study, low molecular weight PEIs were crosslinked with biodegradable disulfide bonds, which were then exploited to enhance their rapid degradation after cellular internalization due to the higher glutathione concentration inside the cells compared to the extracellular environment [94]. In another study, BSA/tetraphenylethylene (TPE)-based quaternary complexes were prepared with cationic amino poly (glycerol methacrylate) derivative (PGMA-EDA) to retain the high transfection efficiency provided by cationic polymers, meanwhile decreasing the cytotoxicity [93]. The obtained polymer/pDNA/TPE/BSA (PDTB) quaternary nanocomplexes demonstrated an enhanced transfection efficiency of 2.5-fold and 4.5-fold to the PEI/DNA binary complexes in A549 and HeLa cells, respectively. Moreover, PDTB nanocomplexes showed lower toxicity in both the cell lines than PEI/DNA, probably because of the decrease in positive surface charge density of cationic polymer after the addition of TPE/BSA NPs.

#### 6.1.2. PEG

The introduction of hydrophilic non-cationic moieties such as polyethylene glycol (PEG) on the albumin nanocarriers prevents the nonspecific protein adsorption, reduces cytotoxicity, inhibits the reticuloendothelial system activation, and increases the half-life in the blood by reducing their phagocytic uptake [95,96]. The first covalent conjugation of PEG to BSA was studied by Abuchowski and co-workers in 1977. They concluded that the PEGylation could significantly decrease immunogenicity, and enhance circulation times and solubility of albumin [97]. Since then, various studies have reported on the surface modification of albumin nanocarriers with PEG for the delivery of nucleic acids in cancer cells [23,71,98]. For instance, Kang and co-workers demonstrated that the conjugation of oligonucleotides to PEGylated albumin prevents oligonucleotide degradation by endonucleases and the unspecific interaction of the nanoparticles with proteins and tissues. Moreover, the RGD peptide on nanoparticles enables the effective delivery of the splice-switching oligonucleotides (SSOs) in human melanoma cells [71]. In other studies, albumin nanocarriers were modified with a heterobifunctional PEG. In this case, one end of the PEG was attached to albumin, and the other end allowed the introduction of new molecules by standard conjugation chemistry approaches [95,98]. Similarly, Kouchakzadeh and co-workers thiolated an antibody (1F2) to facilitate its conjugation to the albumin nanoparticles. In this case, albumin was previously modified with PEG that contains a maleimide group at the outer layer of the structure [98]. Hence, PEG can also be used as a cross-linker for the covalent conjugation of targeting ligands, antibodies, or polymers to albumin nanocarriers.

### 6.2. Targeting Ligands 

The ligand-targeted nanomedicines in oncology involve associating nanocarriers with the molecules that have an affinity to antigens or receptors either overexpressed or uniquely expressed on the target cells compared to the normal cells [99]. The most widely used targeting moieties include peptides, folates, nanobodies, aptamers, and antibodies. The list of albumin-based nanocarriers with various targeting moieties is shown in Table 2. For instance, in a study conducted by Ming and co-workers, splice-switching oligonucleotides (SSOs) were conjugated with RGD (arginine-glycine-aspartic acid) peptide and then to HSA [46]. In contrast, in another study, the RGD-PEG ligand was first conjugated to HSA, which was later conjugated to SSO via reversible S–S bonds [71]. In both cases, there was a receptor-selective delivery of oligonucleotides to the cancer cells. The RGD peptide-modified nanoconjugates showed an enhanced uptake by 61-fold in the A375/Luc705 tumor cells compared to the non-targeted control nanoconjugates [46]. This is because RGD peptide has selectivity for integrin αvβ3, a cell surface glycoprotein, which is overexpressed in angiogenic endothelia and some tumors [100].

#### 6.2.1. Antibodies

The high affinity and specificity of antibodies can be exploited to improve the targeting of different nanocarriers, including albumin-based ones, for tumor cells. These cells often overexpress receptors for peptides, hormones, iron, or folic acids, which can be targeted by antibodies [87]. Among all, monoclonal antibodies (mAb) are the preferred class of targeting molecules. The conjugation of antibodies on the nanocarriers usually enhances their recognition and binding to the targeted cells and their further internalization. In a study by Choi and co-workers, a novel BSA nanocomplex conjugated with anti-ErbB-2 antibodies, harboring Bcl-2-specific siRNA, and gold nanorods, was formulated. The fluorescence-activated cell sorting (FACS) analysis showed five-fold greater internalization of anti-ErbB-2 functionalized BSA nanocomplexes compared to the nonfunctionalized nanocomplexes in SK-BR-3 breast cancer cells, highlighting the advantage of using antibodies. Furthermore, the system was able to induce a synergistic anticancer effect due to the combination of the siRNAs and photothermal therapy mediated by gold nanorods [50].

However, despite the targeting advantages of using antibodies, some challenges are still prevalent, which need to be addressed. mAbs are large and very complex molecules and hence need significant engineering to be effective, their manufacturing is relatively more expensive compared to the small molecules and may increase the size of nanocarriers by up to 40 nm [103].

#### 6.2.2. Aptamers

Aptamers are the novel class of nucleic acid ligands, which are biocompatible, have low immunogenicity, small size, a high binding affinity to the cancer cells, and are easy to modify [20]. Compared to the conventional antibodies used as targeting molecules, aptamers have enhanced tumor/plasma distribution, can better access the solid tumors, and have a facile and scalable synthesis process [104]. There are various classes of aptamers based on their targeting proteins, namely the aptamers targeting vascular endothelial growth factor (VEGF), nucleolin, epidermal growth factor receptor (EGFR), and extracellular matrix (ECM) proteins [20]. Currently, AS1411 is the most investigated aptamer for cancer therapy and is the first anticancer aptamer that reached phase 1 and phase 2 clinical trials [105]. AS1411 aptamer shows high cancer-targeting properties since it has a high affinity to nucleolin, which is overexpressed in the cytoplasm and plasma membrane of cancer cells [20]. However, most of the studies involving aptamers conjugated to albumin nanocarriers are focused on the targeted delivery of chemotherapeutic drugs in cancer. Considering the diverse advantages of conjugating aptamers to nanocarriers, they can be powerful tools for the targeted gene delivery in oncology.

## 7. Conclusions and Future Perspectives

As discussed in this review, albumin nanocarriers have been studied widely for gene therapy in cancer because of the unique features of albumin, such as the ease of preparation, high stability, and biocompatibility. Furthermore, the surface of those nanocarriers can be modified to enhance the therapeutic efficiency and selectivity, whereas reducing the undesired off-target effects. Despite all those features, some limitations are still being reported and need to be addressed properly, such as the albumin catabolism, which may be affected by various factors such as the levels of corticosteroids [65]. Therefore, studies must be conducted to determine the catabolism of albumin in the tumor sites. Moreover, even though albumin is well tolerated and biocompatible, its conjugation with other components presents some safety concerns. For instance, the organic solvents used in the fabrication of albumin nanoparticles, the toxicity profile of cross-linkers such as glutaraldehyde, and compromised stability of albumin during nano-fabrication may hinder the application of these nanocarriers [106,107]. Therefore, further studies are required to ensure the safe use of those nanocarriers to ease their way to the clinic. 

In addition to the prevalent conventional gene therapy, which is mainly focused on the expression of a DNA fragment or its random insertion into the genome, various specific gene-editing tools such as CRISPR/Cas9 have been introduced. These gene-editing tools have promising potential for the introduction of personalized medicines in cancer therapy. In a similar way, novel nucleic acid-based therapies such as chimeric antigen receptor (CAR) T approaches are being developed as a promising therapeutic approach in immuno-oncology. The combination of the advantages imparted by the albumin-based nanocarriers with powerful therapies including CRISPR/Cas9 and CAR-T will revolutionize the treatment options in oncology. Though there are limited studies available on the incorporation of these gene-editing tools in albumin nanostructures, the profound therapeutic application of these vectors is on the near horizon.

## Figures and Tables

**Table 1:** List of albumin-based nanocarriers with nucleic acids for cancer therapy.

Therapeutic Nucleic Acid	Type of Nanocarrier	Size (nm)	Z-Potential (mV)	Model System
**Plasmid**				
Plasmid pORF-hTRAIL (pDNA)	BSA NPs	115.7	−15.4 (pH 7) +11.3 (pH 2)	BALB/c mice bearing i.c. C6 gliomas (Brain Tumor [23])
Plasmid pCMV-EGFP-C	PEI Polyplex	140–450	NA	HeLa cells [44]
hMDA-7 plasmid	BSA NPs	115.6	+33.8	PANC-1 and BXPC-3 human pancreatic cell lines and tumor-induced BALB/c nude mice [24]
pGL3 vector coding for the firefly luciferase gene	HSA-PEI NPs	300 to 700	−7 in H_2_O +16 in 1 mM KCl	Human epithelial kidney 293-cells [45]
**Oligonucleotides**				
Oligonucleotide	Nanoconjugate	13	NA	Tumor spheroids of A375/GFP cells [46]
Antisense Oligonucleotides (ASOs)	HSA NPs	290–330	NA	MCF-7 cells [47]
Akt1 ASOs	Lipid-HSA NPs	108.6	10.5	KB cells and A549 cells [48]
**siRNAs**				
VEGF siRNA	Self-crosslinked HSA NPs	169.3	NA	B16F10 murine melanoma cells, squamous cell carcinoma cells (SCC7), and human prostatic carcinoma cells (PC-3) [49]
Bcl-2-specific siRNA	Anti-ErbB-2 antibody conjugated BSA nanocomplex	278	−39.6	SK-BR-3 and MCF-7 breast cancer cells [50]
phrGFP-targeted siRNA	HSA-coated lipid NPs	79.5	+15.3	MCF-7, MDA-MB-231, SK-BR-3 cells, and phrGFP-transfected MCF-7 xenograft tumor mice model [51]
**Immunotherapeutic biologics**				
Vaccine conjugated with Evans blue (EB) and CpG	Albumin/vaccine nanocomplexes	~13	NA	Female C57BL/6 mice s.c. inoculated with EL4 cells, or EG7.OVA cells, B16F10 cells, MC38 cells on the shoulder [52]
PD-L1 plasmid (CRISPR/Cas9)	Stearyl PEI complexed HSA NPs	203	13	Mouse colon carcinoma CT26 cells [53]

BSA NPs = Bovine Serum Albumin Nanoparticles; HAS = Human Serum Albumin; VEGF = Vascular Endothelial Growth Factor; CRISPR = Clustered, Regularly Interspaced, Short Palindromic Repeat.

**Table 2:** List of albumin-based nanocarriers with targeting moieties for delivery of nucleic acids in cancer therapy.

Targeting Moiety	Description of Nanocarrier	Advantage	Reference
Anti-ErbB-2 antibodies	BSA nanocomplexes with Bcl-2-specific siRNA and gold nanorods	5-fold greater internalization of BSA nanocomplexes	[50]
Folate	Lipid-albumin nanoparticles encapsulating Akt1 ASOs	Provided enhanced selectivity towards folate positive KB cells	[48]
RGD peptide	HSA nanoconjugates with splice-switching oligonucleotides (SSOs)	61-fold enhanced uptake in the A375/Luc705 tumor cells compared to the non-targeted control nanoconjugates	[46]
Trastuzumab	ASOs against Plk1 (polo-like kinase 1) loaded HSA nanoparticles	PlK-1 protein levels were decreased to 46% in BT474 breast cancer cells compared to the controls, while no significant effect was shown with PEGylated albumin nanoparticles	[101]
Anti-EGFR-1 nanobody	Multikinase inhibitor 17864, a platinum-bound sunitinib analog loaded HSA nanoparticles	40-fold higher binding to EGFR-positive 14C squamous head and neck cancer cells in comparison with PEGylated nanoparticles	[102]

ASOs = Antisense Oligonucleotides; RGD = Arginine-Glycine-Aspartic acid; EGFR = Epidermal Growth Factor Receptor; PlK-1 = Polo-like Kinase 1.
